# Entrapment of an air gun pellet between the thyroid cartilage and the lining mucosa in a patient with a penetrating neck injury: a case report

**DOI:** 10.1186/1752-1947-6-184

**Published:** 2012-07-03

**Authors:** Mostafa Hosseini, Mohammad Reza Keramati, Afshin Heidari, Mohammad Kazem Olad-Ghobad

**Affiliations:** 1 Tehran University of Medical Sciences, Rasool-e-akram Hospital, Tehran, Iran; 2 Surgery ward, Tehran University of Medical Sciences, Rasoul-e-Akram Hospital, Sattarkhan Avenue, Tehran, Iran

## Abstract

**Introduction:**

Air guns, either modern or traditional models, are powerful weapons that are capable of causing serious or life-threatening injuries.

**Case presentation:**

Here, we present a case of an air gun pellet injury, with the pellet trapped between the thyroid cartilage and the lining mucosa of a 58-year-old Iranian man.

**Conclusion:**

Entrapment of air gun pellet between thyroid cartilage and the lining mucosa, as presented in our case, may cause diagnostic challenges through the clinical presentation of slight odynophagia.

## Introduction

Modern air guns were invented in the 15th century and came into use during the Napoleonic wars in the late 17th and early 18th centuries. These air guns were designed using an air reservoir connected to a cannon barrel. The devices were capable of propelling a four pound lead ball over a distance of 500 yards, and able to penetrate 3 inch oak board [1]. In contrast, firearms are air pistols that generate more than 8.1 J and air rifles more than 16.2 J. A review of the literature has revealed an alarming trend in increasing incidence and severity of air gun pellet injuries [2].

## Case presentation

**Figure 1:**
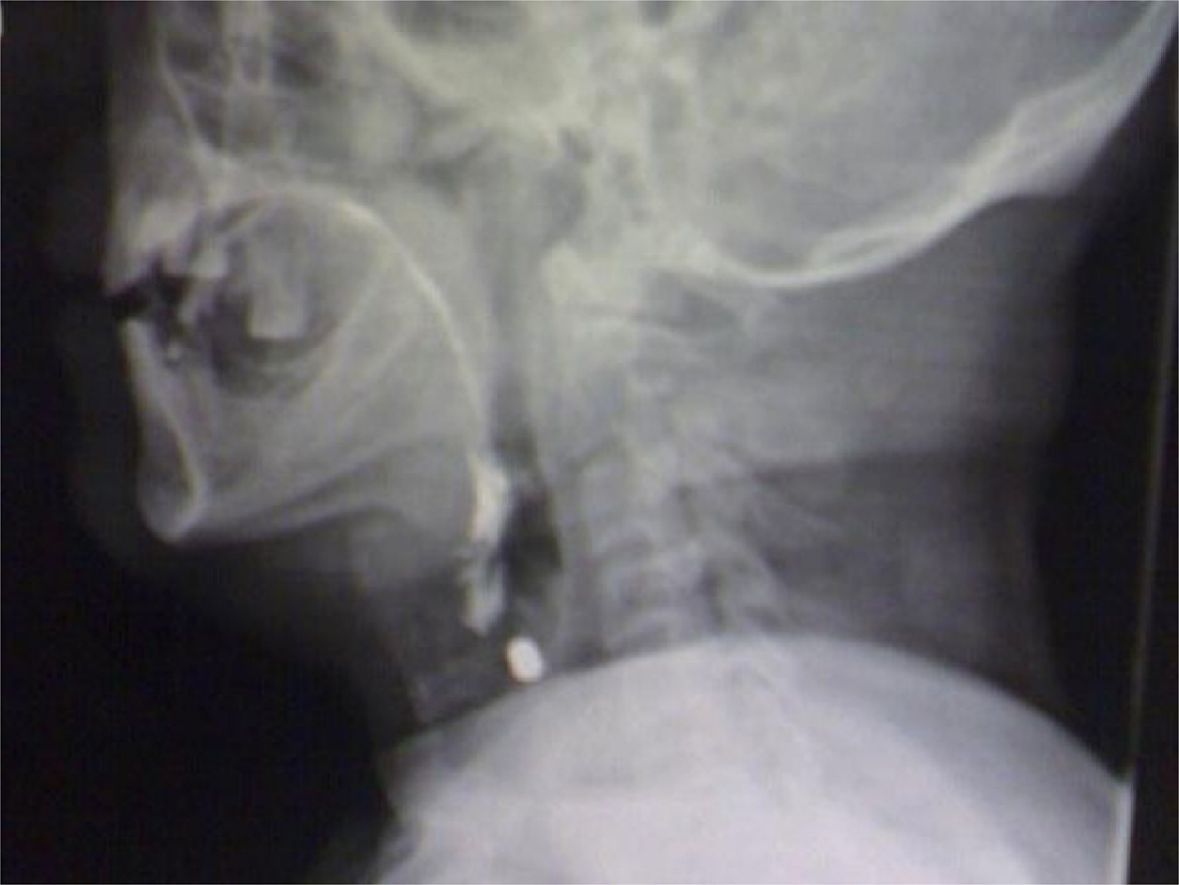
**Plain cervical X-ray (Lateral views) of the patient.** Screening X-rays showed the location of the pellet.

**Figure 2:**
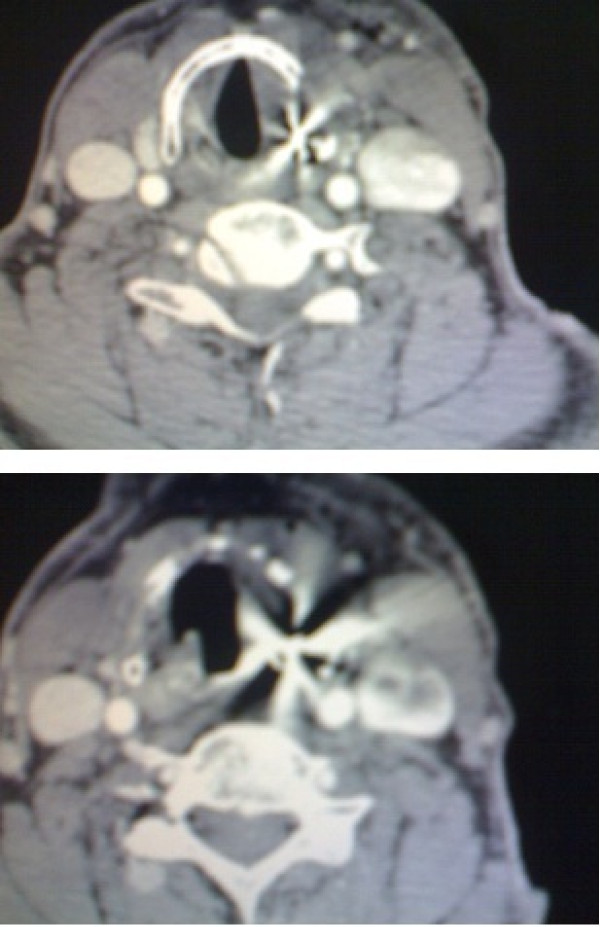
**Computer tomography scan of the neck.** Computed tomography showed the depth of penetration but was not very accurate.

A 58-year-old Iranian man presented to our emergency department after an air gun shot injury. On admission, a penetrating wound in the left lateral region of zone II of his neck was detected. His vital signs were normal. His only complaint was slight odynophagia. No bleeding, hematoma, dysphonia or subcutaneous emphysema was noticeable. On further investigation, anteroposterior and lateral X-rays showed a hyperdense pellet in the left latera region of his neck (Figure 1). A computed tomography scan confirmed the presence of the pellet at the level of the thyroid cartilage (Figure 2).

**Figure 3:**
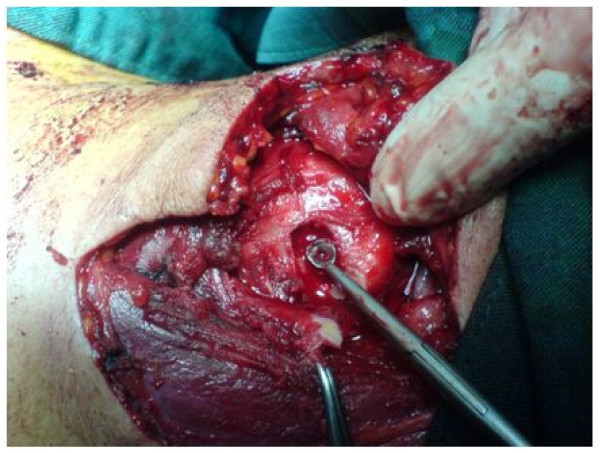
**Intraoperative view of the site of injury.** This figure shows penetration of the cartilage without violating the lining mucosa.

Our patient underwent a neck exploration using a hockey stick incision. After the primary incision of the skin and platysma, the carotid sheath was explored with no findings of note. An intraoperative X-ray of his neck, using control needle markers, revealed a tiny orifice of about 1.5mm on the left superior part of the thyroid cartilage. Extending the orifice to 1.5cm showed a pellet between the cartilage and the lining mucosa (Figure 3). The pellet was removed. After complete repair of the cartilage and placing a drain, the subcutaneous tissue and skin were repaired.

Our patient was discharged after drain removal with good general health. On a two-year follow-up he did not report any problem or complication and his physical examination remained normal.

## Discussion

Air guns, modern or traditional models, are powerful weapons that are capable of causing serious or life-threatening injuries, although modern ones are low-powered due to safety concerns and legal restrictions. The critical velocity required for penetration of human skin by an air rifle pellet is around 125 feet per second to 230ft/s (38 miles per second to 70m/s) [1]. A high energy missile can be defined as an object travelling at a speed in excess of 2,000ft/s. Low energy missile injuries occur at velocities below 1,500ft/s. The velocity alone is not the only factor determining the damage that can be inflicted by an air rifle pellet. The pellet can rapidly lose velocity over distance and thus the pellet velocity at the target is more relevant in terms of tissue damage. Direct effects on tissues occur within the missile tract, such as laceration and crushing, rather than the effects due to temporary cavitation [2].

Most air gun pellet injuries occur in children and adolescents. The majority of fatal incidents reported have involved children under the age of 16, with boys outnumbering girls [3]. The most common site of injury is the head and neck region [2]. Air gun injuries to the eye have been cited in previous reports [4,5]. The airway and neurovascular structures make the neck a potentially life-threatening site of injury, as indicated by a review of the literature. David [6] presented a case of a penetrating air gun injury to the neck, where the pellet was removed from the posterior esophageal wall. Holland et al. [7], in a five-year retrospective study, published two cases of air gun injuries to the neck. Air gun injuries to the carotid arteries have also been studied [2,8]. Cases of retropharyngeal abscess following air gun pellet injuries to the neck have been also reported [9]. Air guns as a method of suicide are unusual, but cases have been reported [3,10].

Air gun injuries may not always be immediately apparent. Patients may be unaware of having been shot and the entry wound is often very small, thus serious injuries may be missed completely [2]. Injuries from high velocity missiles can be entirely different from those caused by low-velocity projectiles. High velocity wounds, particularly when wounds are closed, are dangerous because they may lead to direct or indirect laryngeal and airway obstruction. Additionally, a pellet may embolize within a vessel and be transported to a distant site [2]. Low-velocity injuries are rarely fatal [11,12].

Plain radiographs are important in the evaluation of suspected cases of air gun pellet injuries [2]. Locating air gun pellets using ultrasound-guided techniques can minimize the need for blind exploration of wound tracts. Therefore, theses diagnostic modalities can limit complications such as swelling and hematoma caused by wound exploration [13]. Use of selective angiography in addition to clinical examination has also been proposed by van As et al. [14]. Angiography is a necessity if a missile enters the base of the skull or neck [12].

The necessity of surgical exploration and retrieval of air gun pellets has been debated, particularly where the risks are higher than those associated with leaving the pellet in situ. Most vascular injuries can also be treated by observation [12,14,15].

## Conclusion

Entrapment of an air gun pellet between the thyroid cartilage and the lining mucosa, as presented in our case, may cause a diagnostic challenge through the clinical presentation of slight odynophagia. Odynophagia may distort diagnostic differentials towards esophagus involvement and the trachea may be ignored in such cases.

## Consent

Written informed consent was obtained from the patient for publication of this case report and accompanying images. A copy of the written consent is available for review by the Editor-in-Chief of this journal.

## Competing interests

The authors declare that they have no competing interests.

## Authors’ contributions

“All authors read and approved the final manuscript”. MH was the main supervisor and surgeon. MRK collected the information and complete history of the patient. AH followed the patient through serial examinations and wrote the manuscript. MO-G reviewed the articles and was one of the assistants during the surgery. All authors contributed equally for this case presentation.

## Authors’ information

MH, MRK, AH and MO-G: Tehran University of Medical Sciences. General surgery department, Rasoul-e-Akram Hospital, Surgery ward
